# Understanding mentoring relationships between mentees, peer and senior mentors

**DOI:** 10.1186/s12909-023-04021-w

**Published:** 2023-01-31

**Authors:** Vaishnavi Venktaramana, Yun Ting Ong, Jun Wei Yeo, Anushka Pisupati, Lalit Kumar Radha Krishna

**Affiliations:** 1 Yong Loo Lin School of Medicine, National University of Singapore, 1E Kent Ridge Road NUHS Tower Block Level 11, Singapore, 119228 Singapore; 2 Division of Supportive and Palliative Care, National Cancer Centre Singapore, Level 4, 11 Hospital Crescent, Singapore, 169610 Singapore; 3 Division of Cancer Education, National Cancer Centre Singapore, Level 4, 11 Hospital Crescent, Singapore, 169610 Singapore; 4 Duke-NUS Medical School, Singapore, 8 College Road, Singapore, 169857 Singapore; 5 Palliative Care Institute Liverpool, Academic Palliative & End of Life Care Centre, Cancer Research Centre, University of Liverpool, 200 London Road, Liverpool, L3 9TA UK; 6 Health Data Science, University of Liverpool, Whelan Building The Quadrangle Brownlow Hill, Liverpool, L69 3GB UK; 7 Centre of Biomedical Ethics, National University of Singapore, Blk MD11, 10 Medical Drive, #02-03, Singapore, 117597 Singapore; 8 PalC, The Palliative Care Centre for Excellence in Research and Education, PalC c/o Dover Park Hospice 10 Jalan Tan Tock Seng, Singapore, 308436 Singapore

**Keywords:** Professional identity formation, Mentoring relationships, Palliative medicine, Medical education, Mentoring, Medical students, Physicians, Personhood, Ring theory of personhood

## Abstract

**Background:**

Mentoring relationships play a critical but poorly understood role in mentoring’s overall success. To overcome these knowledge gaps, a study of mentee experiences in the Palliative Medicine Initiative, a structured research-based mentoring program, is proposed. The program’s clearly described mentoring approach, competency-based mentoring stages and curated mentoring environment ensure a consistent mentoring experience. It provides a unique platform to study mentoring relationships longitudinally and its implications on professional identity formation.

**Methodology:**

The Tool Design Systematic Evidence-Based Approach methodology is used to map and employ current understanding. A review of recent reviews on mentoring processes, mentoring’s effects, professional identity formation and professional identity formation assessment tools lay the foundation for the design of semi-structured interviews and mentoring diaries to evaluate the characteristics of successful mentoring relationships and mentoring’s impact on professional identity formation. The data accrued from these tools were evaluated using this methodology whilst changes in professional identity formation were assessed using the Ring Theory of Personhood.

**Results:**

The semi-structured interviews revealed four themes: stakeholders, mentoring stages, mentoring relationships and professional identity formation whilst the mentoring diaries revealed two: mentoring processes and mentoring relationships. Two final domains emerged – mentoring relationships and professional identity formation.

**Conclusions:**

The Palliative Medicine Initiative’s structured stage-based mentoring approach, trained stakeholders, curated environment, assessment-directed and personalized mentoring support reveal seven developmental stages of mentoring relationships. These culminate in changes to the values, beliefs and principles that shape how mentees see, feel and act as professionals. These findings suggest that mentoring programs may help to further develop and fine-tune their professional identity formation.

**Supplementary Information:**

The online version contains supplementary material available at 10.1186/s12909-023-04021-w.

## Introduction

Mentoring’s ability to furnish personalized, holistic, timely and appropriate support cultivates deep and trusting mentoring relationships between mentee, mentor and the host organization (henceforth stakeholders) [1–9]. Sng et al. [10] suggest that it is these mentoring relationships that boost clinical, academic, personal and professional development and influence professional identity formation (PIF) This refers to how mentees see, feel and act as professionals [11–17]. However, a lack of a clear understanding of the nature of mentoring relationships and its role in PIF, as well as varied mentoring practice and a lack of effective longitudinal assessment tools has hampered appreciation of mentoring relationships and its nature [18–20]. This has compromised design of mentoring programs and efforts to employ mentoring to guide PIF.

### The Palliative Medicine Initiative

The Palliative Medicine Initiative (PMI) hosted by the Divisions of Supportive and Palliative Care (DSPC) and Cancer Education (DCE) at the National Cancer Centre Singapore (NCCS) provides an opportunity to study mentoring relationships in a structured research mentoring program and curated mentoring environment [7, 9, 21]. The PMI’s use of Combined Novice, Peer and E-mentoring approach or CNEP mentoring offers a stage-based mentoring approach that facilitates longitudinal study of interactions between various stakeholders [1, 7, 22–28].

Within the PMI, the host organization oversees and structures the mentoring program. Senior mentors are trained clinicians, well-versed in the CNEP mentoring approach, and experienced in research mentoring and medical education. In turn, peer mentors are experienced mentees who have successfully completed at least one PMI project and trained to provide complementary mentoring support to new mentees.

Just as the PMI offers a unique opportunity to study mentoring relationships within a structured mentoring program, recent developments in the study of moral distress [29], dignity [30, 31], how physicians, nurses and medical students cope with death and dying [32–35] and reviews into assessments of PIF [16, 18, 36] allow the employ of the Ring Theory of Personhood.

### The Ring Theory of Personhood (RToP)

Underpinning the utilization of a RToP-based tool is the notion that how a mentee feels, acts, and sees themselves as professionals and in their mentoring roles is shaped by their beliefs, values and principles (henceforth the belief system). This belief system is said to be fashioned by their self-concepts of personhood [37]. It is also influenced by their experiences, personal narratives, contextual considerations and growing clinical competence.

**Fig. 1:**
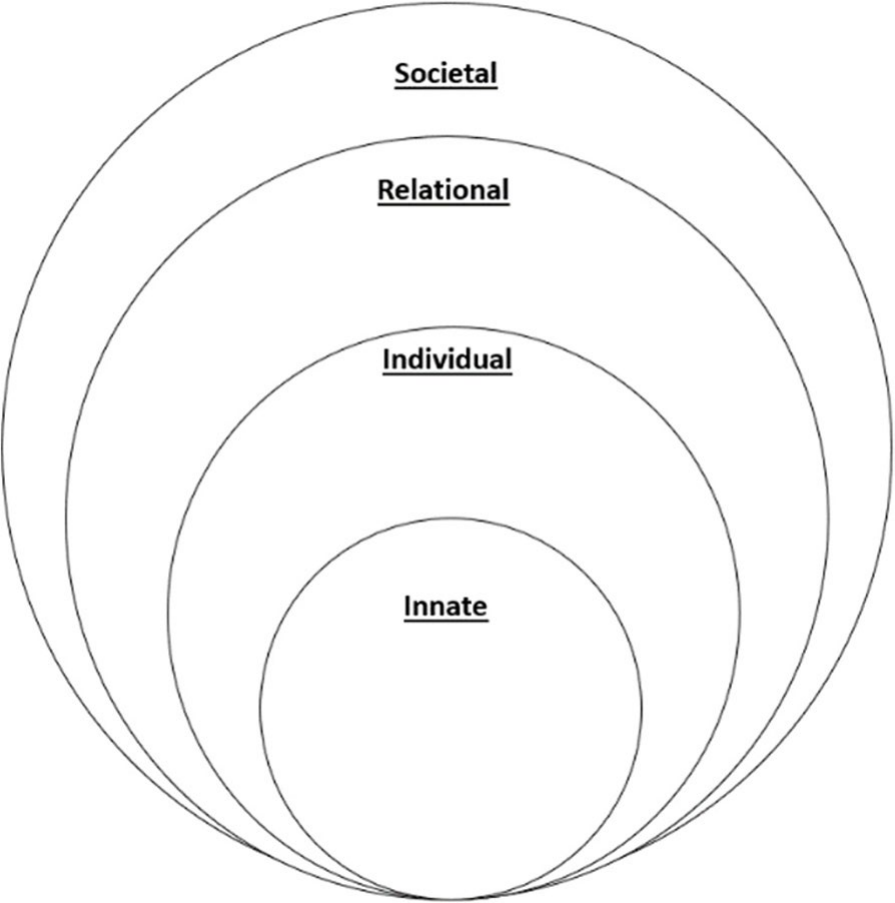
The Ring Theory of Personhood

There is growing evidence that Krishna’s Ring Theory of Personhood is able to map changes in self-concepts of personhood and identity. Such discernment requires careful appreciation of competing considerations shaping the mentee’s response to their mentoring, clinical, personal and research experiences. To this end, each of the RToP’s Innate, Individual, Relational and Societal Rings captures specific aspects of the mentee’s belief system (Fig. 1).

The Innate Ring’s belief system is rooted in spiritual, religious, moral, and ethical values, beliefs, and principles. The Individual Ring’s belief system draws on elements of conscious function. The Relational Ring’s belief system is fashioned by the values, beliefs, and principles that underpin the mentee’s personal and important relationships. The belief system within the Societal ring is structured by sociocultural, professional, organizational, clinical, ethical and legal influences within the wider community.

These belief systems evolve as a mentee progresses through the mentoring program. When encountering experiences that are consistent with the mentee’s belief system there is ‘resonance’. When ‘resonant’ belief systems within one or more rings are adapted to better align with current practice, there is ‘synchrony’. However when experiences are in conflict with the belief system in one ring, there is ‘disharmony’. If these conflicts with regnant belief systems extend to more than one ring there is ‘dyssynchrony’. Appreciating the presence of resonance, synchrony, disharmony and dyssynchrony and their effects provides a means of mapping changing concepts of identity with PIF.

## Methodology

The research questions, “*What are the characteristics of a successful mentoring relationship*?” and “*What impact does mentoring have on Professional Identity Formation (PIF)?”,* necessitates the design of a PIF assessment tool.

### Systematic Evidence Based Approach (SEBA)

**Fig. 2:**
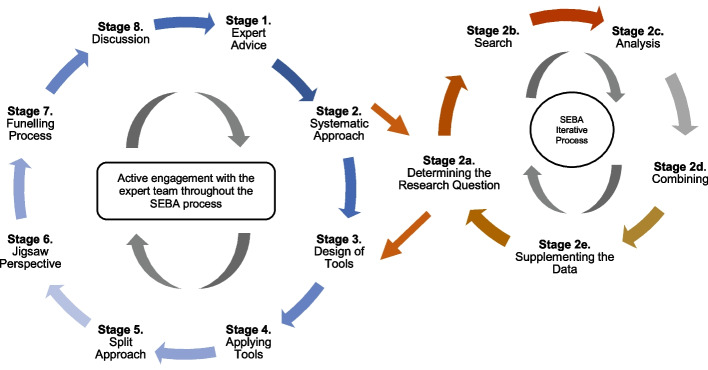
The Tool Design SEBA Process

The Tool design SEBA methodology designed around Krishna’s Systematic Evidence Based Approach (SEBA) was adopted to guide this two-staged study (Fig. 2) [1, 4, 8, 19, 21, 29–32, 36, 38–42]. The Tool design SEBA methodology is detailed in Appendix 1.

### Stage 1. Expert team advice

An expert team of experienced researchers guide the research team and oversee the stages of the Tool Design SEBA methodology.

### Stage 2: Tool design

Tool design will be informed by a review of current reviews of mentoring and PIF. The detail of this SEBA guided process is provided in Appendix 1.

### Stage 3: Design of interviews and diaries

The combined information from the SSRs in SEBA were supplemented with data from earlier studies on the PMI to contextualize the tool design. The design of the semi-structured interviews and mentoring diaries were also guided by a review of Teo et al. [36]‘s review of PIF assessment tools.

### Stage 4. Conducting interviews and diaries

Purposive sampling of PMI mentees was conducted and the participant information sheet containing the information on the aims of the study, and the participant’s rights to privacy, anonymity and to withdraw from the study at any point without prejudice were included in the email invite. The 30–45 min audio-recorded semi-structured interviews conducted over the Zoom video conferencing platform were carried out between February and May 2021 by experienced and trained interviewers, AP and CQWL. The mentoring diaries were conducted on Google Forms and were completed between March to December 2021.

Ethics approval (reference number: 202010–00084 and 202,103–00057) was obtained from the Singhealth Combined Institutional Review Board. Informed written and oral consent was obtained from all the participants.

### Stage 5. Split approach

Three independent teams, each guided by a senior trained PMI mentor carried out the analysis of the anonymized data. The first team thematically analyzed the transcripts of the semi-structured interviews using Braun and Clarke [43]‘s approach to thematic analysis. Using Hsieh and Shannon [44]‘s approach to Directed Content Analysis, the second team drew categories for the analysis from Krishna and Alsuwaigh [37]‘s article entitled “U*nderstanding the fluid nature of personhood - the ring theory of personhood*” and Kuek, Ngiam [45] entitled “*The impact of caring for dying patients in intensive care units on a physician’s personhood: a systematic scoping review*”. The third team carried out thematic and content analysis of the mentoring diaries.

Each team carried out regular online discussions during the coding process and used Sandelowski and Barroso [46]‘s approach to ‘negotiated consensual validation’ to reach consensus on the codes identified. As the coding process was a training process overseen by mentors and the expert team, Kappa inter-reliability scores were not evaluated.

### Stage 6. Jigsaw perspective

This process combines overlapping themes and categories to create themes/categories.

### Stage 7. Funneling process

The themes/categories from the mentoring diaries and interviews were combined to create domains that frame the discussion [47].

## Results

The themes/categories drawn from the Jigsaw Perspective involving the 12 semi-structured interviews and 17 diaries are presented separately to enhance transparency (Table 1).

**Table 1:** Themes/Categories Identified in Interviews and Diaries

Semi-structured Interviews	Mentoring Diaries
1. Stakeholders ▪ Interactions between mentee, mentor, peer-mentor, and the host organization 2. Stages of Mentoring ▪ Includes the pre-mentoring, initial research meetings, data gathering, review of initial findings, manuscript preparations and reflections stages, 3. Mentoring Relationships ▪ Characterizing mentoring relationships and its effects upon the mentee’s research, personal, academic and career considerations 4. The Ring Theory of Personhood ▪ Delineating shifts in the mentee’s Innate, Individual, Relational and Societal Rings brought on by dissonance, dyssynchrony, disharmony and resonance	1. Stakeholders ▪ Interactions between mentee, mentor, peer-mentor, and the host organization 2. Mentoring Relationships ▪ Characterizing mentoring relationships and its effects upon the mentee’s research, personal, academic and career considerations

The Funneling Process revealed two domains delineating 1) the development of mentoring relationships thorough the mentoring stages and 2) the development of PIF through the lens of the RToP.

### DOMAIN 1. Mentoring relationships through the mentoring stages

This domain concerns the mentee’s perception of the development of their mentoring relationship through the mentoring stages.

### Pre-mentoring stage

At the pre-mentoring stage, mentees were introduced to the PMI’s goals, expectations, codes of conduct, structure, mentoring approach, culture, and support mechanisms, as the host organization determined whether the mentee’s abilities, motivations, availabilities and personality were consistent with the program’s goals and ethos. Prospective mentees revealed that mentors emphasized the program’s shared desire to “pay it forward… that really resonated [with] me” (M9). Mentees were motivated to participate for the opportunity to be mentored along research process (M2) as the PMI would “hold your hand and walk through the entire research process” (M12).

### Initial meeting stage

This stage sees the initiation of the mentoring relationship between the host organization, mentor (henceforth SM), peer-mentor and the mentee.

Mentees were matched to appropriate projects depending on their interests, project availability and SM’s research interests. The project’s learning outcomes, timelines, and deliverables as well as personalized project specific goals, learning objectives, assessment timepoints and endpoints were discussed and agreed upon. Mentee (M11) noted that mentors provided “a succinct explanation of what the project was, and what my learning outcomes [would be]…a very broad overview of what was expected of me, and what I would learn.” Mentee (M1) saw these initial interactions as the start of a personalized mentoring relationships stating “he makes the effort to get to know you better... he took the first steps in rapport building.”

### Data gathering stage

This stage saw mentored immersion into the research process with new mentees supported by their peer-mentors and SMs. Whilst being the most intense and longest stage of the research process SMs and peer-mentors were accessible and provided individualized support “if you needed any help [with] even the smallest questions, they wouldn’t deem it as a stupid question” (M4). This helped mentees overcome their initial anxieties and lack of knowledge, skills and confidence (M12).

Mentoring support was also

- **Timely:** “They were very open to answering and they gave super prompt replies.” (M4)
- **Responsive**: “It just piled up after a while but…there was a lot of guidance along the way.” (M1)
- **Accessible:** “As busy as [the SM] is, he always makes himself available for his students.” (MD7)

These features supported the mentee’s assumption of new responsibilities in the mentoring project.

### Review of initial findings stage

This stage saw mentees undertaking data analysis and synthesis and more active role in the projects shifting from “They told me to…” to “I did/suggested…” statements (M5). This process was supported by mentors who “contributed to greater confidence, greater belief in my own capabilities, and what I can do as a medical student” (M5) and who role modelled positive practices (MD2).

### Manuscript preparation stage

Active participation in the program developed still further in the manuscript preparation stage with mentee (M7) stating “It was like pitching to the SM my original idea for the paper.”

During this stage, communication remained personalized and adaptive to the mentee’s needs: “It [interactions with the mentor and peer-mentor] is just right and it gives me the space and time to work…at the same time [I am able to] check in with him to make sure that I am on the right path.” (MD14). This facilitated deeper connections with mentees seeking guidance in non-research related areas and personal issues (MD17).

### Reflections stage

Reflecting upon their PMI experiences, mentees reported a sense of pride in the personal and professional development (M2*)* and in their personal contribution to clinical practice (M7). It also allowed mentees to appreciate “what good mentoring [was] like” (M2), where free communication (M7) and a non-judgmental learning environment were provided (M4) and reflect on its effects on their development (M4). Mentees also described taking on the values espoused by their mentors such as “personal management, self -discipline.” (M5) and adopt new practices such as how they interact with their juniors and how they “build rapport.” (M1).

Some negative experiences that were recounted include mismatched expectations (M5, M6), poor communications (M2, M5, M6, M12), limited guidance (M2, M10) from busy clinicians, and ‘stunted’ mentoring relationships due to large group sizes (M5, M6).

### DOMAIN 2. Development of professional identity formation through the lens of the RToP

Seen through the RToP the mentoring process provides a longitudinal perspective of professional identity formation (PIF) and the impact of resonance, synchrony, disharmony and dissonance.

### Resonance

- **Innate Ring:** There were shifts in perspectives on spirituality and/or their moral compass – “I’m a Buddhist and [the research mentoring journey] did reinforce some of the philosophies behind it.” (M2)
- **Individual Ring:** Mentees re-evaluated their values, beliefs, hopes, self-expression, and self-awareness – “I started to ask myself…What were my qualities? What do I want in life?” (M7)
- **Relational Ring:** Mentees reported changes in how they interact with their families especially in the context of end-of-life care – “[It] made me think about how I should start planning…myself and my family members, what would they want out of their care?” (M7)
- **Societal Ring:** Mentees were able to apply their learning to their professional roles as medical professionals – “When I saw what the Senior Mentor was like, I was thinking, that’s the kind of doctor I want to be that kind of level of EQ and level of interaction with patients.”(M10)

### Synchrony

- **Between Societal and Relational Rings:** “Learning how to draw boundaries reasonably and communicating effectively…extended to my personal relationships as well within my family... it’s made the relationship a lot healthier…” (M1)
- **Between Societal and Innate Rings:** “Learning about death and dying patients, and the process at the end of life…reinforced my religious beliefs.” (M2)
- **Between Societal and Individual Rings:** “I wouldn’t say [beliefs about my work and career] changed dramatically…it reaffirmed and consolidated what I knew about myself.” (M10)
- **Between Individual and Relational Rings**: “Now I’m thinking about things maybe in terms of relational aspects” (M7)

### Dyssynchrony

Dyssynchrony between societal and individual ring saw challenges in striking a healthy work-life balance: “It’s hard (to) manage your time with it when you have other commitments as well… that’s really a compromise and sleep, your health, your social life and your schoolwork.” (M6).

### Disharmony

Disharmony was especially evident within the societal ring. This saw differing views on responsibilities. “I think a good doctor needs to be able to connect with patients and understand them. But there isn’t that obligation to teach [this in school].” (M12) In addition, the medical hierarchy led to difficulties in connecting with the younger mentees: “How I actually interact with my juniors...is affected by a sort of power imbalance in a sense and sometimes the juniors may not be ready [for it].” (M1).

## Discussion

### Stage 8. Discussion synthesis

In answering the question, “*What are the characteristics of a successful mentoring relationship?*”, this study reveals seven stages in the development of mentoring relationships.

Stage one concerns the mentoring relationship platform. This platform is shaped by the mentoring framework employed by the host organization. The mentoring framework maps the mentoring curriculum, process, approach, assessments and support mechanisms. Given the PMI’s goal of guiding mentees towards primary authorship in research publications, the mentoring framework encourages mentees to move from a peripheral to a more central role in the research process by providing them with training, guidance, feedback and support.

Stage two sees the integration of support mechanisms within the mentoring relationship platform. This includes robust and accessible two-way open communication channels, protected time for mentoring and ‘goodness of fit’. ‘Goodness of fit’ requires tailoring the mentoring approach, working styles, timelines, expectations, roles, responsibilities and meeting schedules to accommodate the mentee’s changing goals, needs, abilities, experience and availabilities.

Stage three focuses on nurturing enduring mentoring ties through adaptable and personalized support. Responsive, timely, accessible, holistic, and longitudinal support by trained mentors introduce ‘flexibility’ to the mentoring framework. ‘Balancing’ ensures that flexibility does not compromise compliance to parameters set out by codes of conduct.

**Fig. 3:**
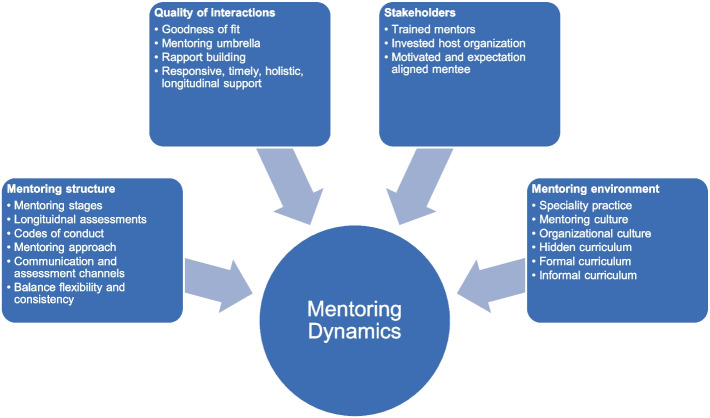
An Evidence Based Evolution on the Concept of Mentoring Dynamics

Stage four highlights the importance of the mentoring dynamics between stakeholders. These dynamics reflect the quality of mentoring interactions (Fig. 3).

**Fig. 4:**
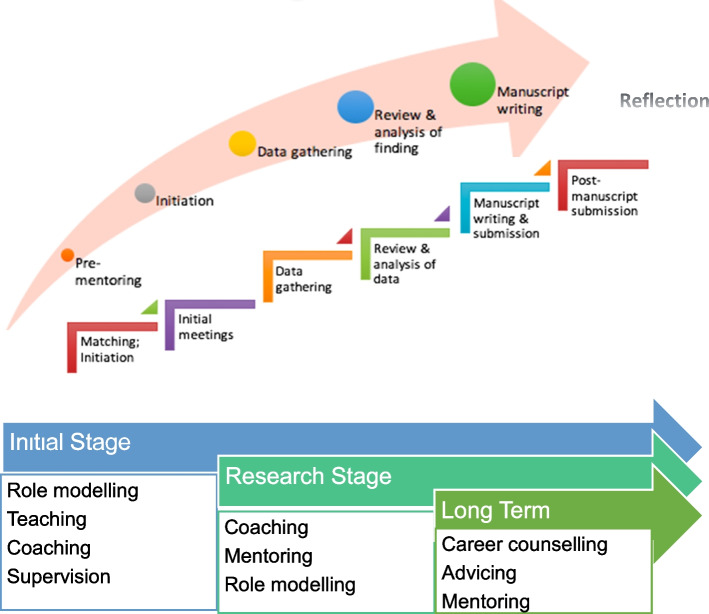
Support Along the PMI Stages

Mentoring dynamics pivot on the personalization of mentoring relationships provided by the ‘mentoring umbrella’. In the initial mentoring stages where role modelling, teaching, and coaching personalize the mentoring experience, counselling and advising later give way to assessment-based coaching and project supervision (Fig. 4). This mix of support mechanisms boosts the quality of mentoring interactions, develops the mentoring relationship, and extends interactions to include the provision of personal and career advice and support.

Stage five assesses the mentoring relationship. This is done at each mentoring stage and considers evolutions in the mentee’s beliefs system, practice and outlook.

Stage six sees the employ of assessment data to guide mentoring support and shape adaptations to the mentoring approach.

**Fig. 5:**
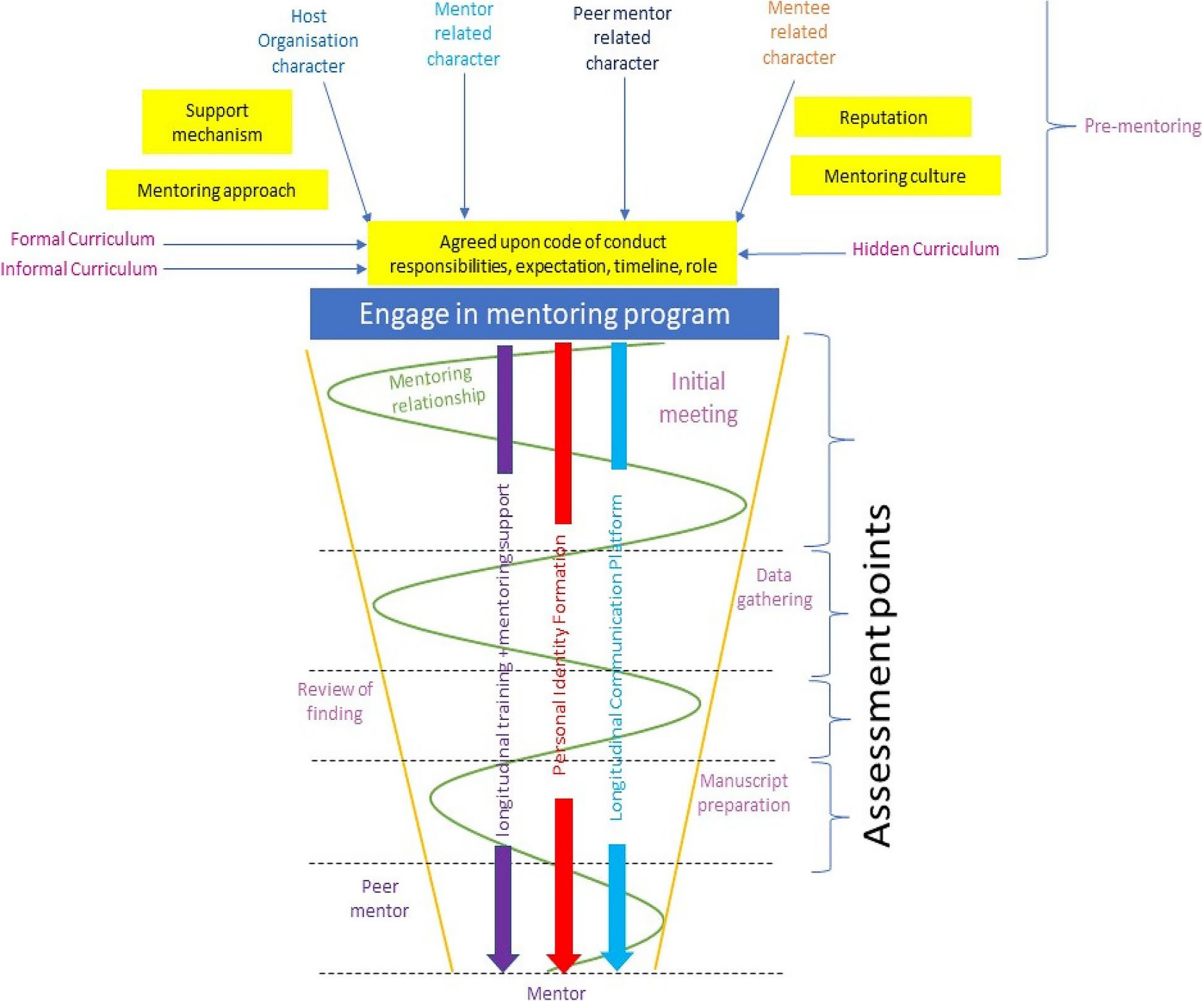
The Mentoring Structure, its Key Elements and its Intertwined Relationships

Stage seven helps address the research question “*What impact does mentoring have on Professional Identity Formation (PIF)?”*. In their mentoring diaries, mentees suggest that a change occurs in the manner they feel, see and act about their role in the PMI as mentors guide and role model pertinent values, beliefs and principles to the mentees, tutor them in the appropriate knowledge and competencies required, coach them in research skills and team-based practice, supervise them throughout the research process and direct longitudinal and personalized mentoring support within the confines of the mentoring framework. These features provide evidence of mentoring’s impact upon identity formation. These features are summarized in Fig. 5.

Progress through the mentoring stages reveals the presence of synchrony, resonance, disharmony and dyssynchrony. This reflects the impact of new practices, values, beliefs and principles upon the mentee’s current belief system.

These changes affirm mentoring’s impact on PIF. Figure 5 also hints at possible mechanisms behind the PMI’s ability to nurture PIF. “Acculturation into, and identification with” the PMI program suggests that the socialization process occurs within PMI [48, 49] – a process “by which a person learns to function within a particular society or group by internalizing its values and norms”. Given its structured approach in funneling mentees from peripheral participation to central roles via mentored immersion and a nurturing mentoring environment, mentoring programs may indeed serve as a community of practice for medical students and physicians, “a persistent, sustaining social network of individuals who share and develop an overlapping knowledge base, set of beliefs, values, history and experiences focused on a common practice and/or enterprise”, a place for them to develop and fine-tune their professional identity.

### Limitations

Whilst the data accrued from the semi-structured interviews and mentoring diaries were drawn from 29 mentees and echoed the findings of large-scale reviews on mentoring relationships, programs and the mentoring environment, the mentees were interviewed at a single time point, often after completion of a project or at the later stages of their research process. This may have led to recall bias and the halo effect. Whilst the use of diaries to triangulate the interview data added depth to the analysis, there were limited entries.

## Conclusion

The Palliative Medicine Initiative’s structured stage-based mentoring approach, trained stakeholders, curated environment, assessment-directed and personalized mentoring support reveal seven developmental stages of mentoring relationships. These culminate in changes to the values, beliefs and principles that shape how mentees see, feel and act as professionals. These findings suggest that structured mentoring programs may help to further develop and fine-tune their professional identity formation. We posit that portfolios containing mentoring diaries, reflections and regular assessments of their mentoring progress could provide a better appreciation of mentoring’s relationship with professional identity formation.

## Supplementary Information

**Additional file 1.**

## Data Availability

All data generated or analysed during this study are included in this published article.
