# Supplementary material for: Understanding mentoring relationships between mentees, peer and senior mentors
Source: BMC Med Educ. 2023 Jan 31;23:76. doi: 10.1186/s12909-023-04021-w (PMC9887801; doi:10.1186/s12909-023-04021-w)
Supplement: Supplementary file 1 — Additional file 1. [file 12909_2023_4021_MOESM1_ESM.pdf]

Appendix 1.
SEBA PROCESS

Theoretical lens

The Tool design SEBA methodology's Constructivist ontological and Relativist epistemological traditions are consistent with Constructivist concepts (1) behind regnant PIF theories and the notion that PIF and self-concepts of identity and personhood are fluid sociocultural and interpersonal constructs informed by the peer-mentor's narratives, contextual and environmental considerations (2). This lens also suits the adoption of the RToP for the study of long-term developmental changes and short term-contextual fluctuations spurred by synchrony, resonance, disharmony, and/or dyssynchrony.

Stage 1. Expert advice

An expert team consisting of a medical librarian from the Yong Loo Lin School of Medicine (YLLSoM) at the National University of Singapore (NUS), and local educational experts and clinicians at the National Cancer Centre Singapore (NCCS), the Palliative Care Unit, and the Institute of Population Health at the University of Liverpool, YLLSoM and Duke-NUS Medical School, ensured that the SEBA methodology was employed in a consistent manner within accepted practices. This was especially important given that the SEBA methodology was being employed to guide scoping reviews that will inform the design of semi-structured interviews and the mentoring diaries, and the analysis and comparison of the data collected from these tools.

Reflexivity

Personal reflexivity

Recognising that the research team are informed by their interests, narratives, clinical and research insights and contextual considerations, membership to the research and expert teams were made up of experienced physician-tutors, psychologists, methodologists, and educational scholars. Six members of these teams held masters degrees in medical education, two held masters degrees in ethics, two held masters degrees in clinical research and two hold masters degrees in public health. Most of the expert and research team were experienced in quantitative research with five members of the team were experienced in qualitative methods. Six have published articles in peer-reviewed journals using the SEBA methodology. One member of the expert team is an experienced researcher at the Palliative Care Institute Liverpool and another is a member of the Health Data Science Department at the

University of Liverpool. Both researchers from the University of Liverpool have been collaborating with the research team on a number of studies pertaining to death and dying, moral distress, PIF and mentoring and are part of the team expanding the use of the SEBA methodology in medical education and palliative care.

Three senior members of the team are working on various projects on PIF and three others have already published articles on PIF. Six members of the research team were members of the PMI and have published on mentoring practice. To ensure input from all members of the team, synchronous and asynchronous in-person and online meetings and Sandelowski and Barroso (3)'s approach to 'negotiated consensual validation' was used to reach consensus on the issues discussed.

Methodological reflexivity

Adopting a structured constructivist approach, we sought to build a holistic concept of current models of PIF within medical education acknowledging however that we were limited by manpower and time constraints. We included articles featured in grey and bibliographic databases within the dates set in our selection criteria. Much of these theories required deeper consideration and discussions and we documented our discussions and decisions.

Stage 2: Systematic approach

To address "*What are the characteristics of a successful mentoring relationship*?" and "*What impact does mentoring have on Professional Identity Formation (PIF)?"* the research and expert teams adopted the Tool design SEBA methodology designed around Krishna's Systematic Evidence Based Approach (SEBA) to guide this two-staged study (Figure 2).

Stage One involved design of a PIF assessment tool for the mentoring setting given the absence of such a tool in mentoring practice. Stage Two involves employ and analyse the data drawn from the tools designed.

Stage 2a. Determining the Research Question

In truth, there are two elements to Stage One. The first revolves around understanding "What are the characteristics of a successful mentoring relationship?" the second part is considering "*how should PIF be assessed?".* Given that both these topics have been covered by recent reviews and supplemented by updates, we will draw upon data from current reviews.

Before we proceed, we provide definitions of some of the terms that will be evaluated.

**Practice**	**Definition from Radha Krishna *et al.* Educational roles as a continuum of mentoring's role in medicine – a systematic review and thematic analysis of educational studies from 2000 to 2018. (4)**
Role model	"A process where a trainer consciously or unconsciously demonstrates positive or negative behaviours, actions or attitudes. The learner observes, weighs up and reflects upon these characteristics, skills and or behaviours upon their own practice/attitude/behaviour and emulates, experiments, and assimilates it into his/her own personal/professional identity. Positive role modelling is more impactful when it occurs in a trusting, professional relationship."
Teaching/ Tutoring	A professional goal-specific, task-oriented, standardized, and structured learning process on clinical knowledge and skills driven by clinical competency and performance outcomes. The professional tutor-, and student-dependent tutor-learner relationship requires protected time to develop in a safe and productive learning environment, supported by the host organization for effective teaching and feedback processes."
Coaching	Longitudinal professional relationship between an expert coach and a trainee focused Upon mastery of a clearly defined, measurable and achievable skill that is that the trainee or training organization feels the trainee can improve upon. The relationship is built upon professional trust in a 'safe environment' that facilitates practice of the skill. The coach evaluates the performance, needs and abilities of the trainee, role models skills, encourages learning, provides specific individualized feedback and devises a plan to achieve the goals. The trainee is accountable for their training and responsible for self-monitoring."
Supervision	"Individualized, focused, goal-specific, time-limited and context-sensitive clinical training process by a senior clinician aimed at assessing and improving particular gaps and weaknesses in the clinical care and patient safety by trainees by providing them with oversight, guidance and feedback and holding trainees up and accountable to established clinical standards and codes of practice
Novice mentoring	"The process of creating personalized, enduring, and mutually beneficial mentoring relationships between stakeholders. Its success necessitates the guidance of a mentoring structure that is able to balance demands for flexibility in accommodating for evolving mentoring needs, goals, circumstances of stakeholders, and the mentoring environment yet maintain a consistent mentoring approach that exists within the confines of codes, standards of practice, and program expectations. To support novice mentoring's dynamic, entwined, evolving, adaptable, context-specific, goal-sensitive, mentee-, mentor-, host organization-, mentoring environment-, mentoring approach-, and mentoring relationship-dependent nature, the host organization must oversee, assess, and support the mentees, mentors, matching process, mentoring relationship, mentoring approach, and mentoring environment."

Stage 2b. Search

The reviews included were drawn from reviews carried out by the PMI and do capture current thinking on mentoring (Table A) and PIF (Table B). The lessons learnt from the study of these included reviews are summarised in Tables A and B

Mentoring

Study of current data on mentoring relationships focused on recently published accounts on the PMI framework (5-8), mentoring programs (9-13), mentoring practice (14, 15), mentoring assessments (16-20), the mentoring environment (12) and the influence of the host organisation on the mentoring process (20). These represented the most recent reviews on the key aspects of mentoring.

Table A. Current reviews on mentoring

Author	Publication	Lessons drawn
**Mentoring relationships**		
Sng et al (2017) (21)	Mentoring relationships between senior physicians and junior doctors and/or medical students: A thematic review.	1. Mentoring relationships evolve in relatively consistent stages<br>2. Mentoring relationships are individualised; stakeholder-, context-, relational-dependent, stage specific<br>3. Relies on timely, personalised, appropriate, holistic, longitudinal support
Krishna et al (2020) (22)	Enhancing mentoring in palliative care: an evidence based mentoring framework.	1. Mentoring's dynamic, entwined, evolving, adaptable, context-specific, goal-sensitive, mentee-, mentor-, host organization-, mentoring environment-, mentoring approach-, and mentoring relationship-dependent nature,<br>2. The host organization must oversee, assess, and support the mentees, mentors, matching process, mentoring relationship, mentoring approach, and mentoring environment.<br>3. Mentoring dynamics refer to the quality of interactions between stakeholders. It is critical to the development of mentoring relationships is influenced by particularly influenced by the mentoring culture<br>4. Built on mentoring stages<br>5. Mentoring relationships pivot on a mentoring ecosystem containing stakeholder-led microenvironments and a macro-environment containing the program and organization culture and curriculum<br>6. Competency based mentoring stages allow assessments to guide practice and support. Each mentoring stage has a core competency that must be met before progressing to the next stage of the mentoring process
**Mentoring approach**		
Toh et al (2022) (23)	The role of mentoring, supervision, coaching, teaching and instruction on professional identity formation: a systematic scoping review.	1. Mentoring's ability to nurture effective mentoring relationships and PIF revolves around its combine supervision, coaching, tutoring, instruction, and teaching and mentoring approach<br>2. The mentoring umbrella provides longitudinal, adaptable, appropriate, personalised support<br>3. The process relies on a common understanding and understanding of the key aspects of knowledge, skills and an appreciation of the codes of conduct<br>4. Alignment of expectations<br>5. A consistent structure and responsive communication, support, assessment and oversight processes support the socialisation process<br>6. Socialisation process is overseen and guided by the host organization process that ensure appropriate structure and support

Krishna et al (2019) (4)	Educational roles as a continuum of mentoring's role in medicine – a systematic review and thematic analysis of educational studies from 2000 to 2018.	1. Role modelling -experiential learning and in the socialisation process. Maybe purposive or opportunistic source of sharing personal/professional practice/skills<br>2. Teaching/tutoring – experiential learning, discussions and guided reflections and nurtures educational relationships<br>3. Coaching – duration time and focus on specified skills. Relies on safe environment and trusting relationships<br>4. Supervision – hierarchical
Lim et al (2020) (15)	Enhancing geriatric oncology training through a combination of novice mentoring and peer and near-peer mentoring: A thematic analysis of mentoring in medicine between 2000 and 2017.	1. Mentoring success depends on<br>• Consistent mentoring approach<br>• Effective mentee recruitment, training, participation and support<br>• Mentor recruitment and training<br>• Acknowledgement of mentor contributions<br>• Effective and consistent mentoring stages
**Mentoring structure**		
Chia et al (2020) (24)	The pivotal role of host organizations in enhancing mentoring in internal medicine: a scoping review.	1. Core roles include<br>• establishing and/or complying with overarching goals, clinical standards, and curriculum requirements<br>• designing, influencing, and overseeing the mentoring program<br>• and nurturing the mentoring culture and mentoring relationships<br>2. Secondary roles include<br>• provide consistent leadership<br>• proactively support mentor and mentee participation<br>• cogently facilitate all mentoring processes<br>• proactively gather and revert feedback on the mentoring program and the mentoring relationships within<br>• and, finally, successfully initiate curricular reform to better meet the needs of their participants<br>3. Ensuring balance between flexibility and consistency in the mentoring approach and structure
Hee et al (2020) (19)	The development and design of a framework to match mentees and mentors through a systematic review and thematic analysis of mentoring programs between 2000 and 2015.	Matching enhances 'goodness of fit
Goh et al (2022) (25)	Mentoring in palliative medicine in the time of covid-19: a systematic scoping review.	1. Requires a non-hierarchical approach<br>2. Relies on mentoring stages<br>3. Overseen and structured by host organization<br>4. Requires longitudinal and holistic assessments – possibly through use of portfolio
**Mentoring Environment**		
Hee et al (2019) (26)	Understanding the mentoring environment through thematic analysis of the learning environment in medicine.	1. The learning and mentoring environment may be studied together<br>2. The learning environment consists of structure and culture.<br>3. LE is the "product of the culture and structure shaped by evolving interactions between the tutor, the learner, the host organization and the formal, informal and hidden curriculum (henceforth quartet of stakeholders). The structural element of the LE influences the quality of learning, supports the personal and professional development of the learner and the tutor and ensures that learning interactions remain within the confined accepted codes of conduct and professional standards of practice. Cultural considerations help personalize the LE and meet the unique and evolving needs of the

		quartet of stakeholders. These factors highlight LE's evolving, goal-sensitive, context-specific, learner-, tutor-, relational-, organization-dependent nature (LE's nature). It forms part of the holistic evaluations to be considered when balancing the sometimes-competing demands for consistency and flexibility in the LE"<br>4. Structure refers to the framework that shapes the learning approach and ensures consistent professional and personal support for learners and tutors within the programme.<br>5. Structure is shaped by the formal curriculum, the stakeholders and the mentoring relationship<br>6. Culture refers to the norms, values, beliefs, practices and support moulding the socioemotional environment in which learning occurs<br>7. Culture is informed by the hidden and informal curricula, the stakeholders and their relationships
**Assessing mentoring**		
Ng et al (2020) (27)	Assessing mentoring: A scoping review of mentoring assessment tools in internal medicine between 1990 and 2019.	1. Most tools do not consider mentoring's<br>• evolving nature<br>• longitudinal effects<br>• stakeholder-dependent features<br>• the wider environmental influences<br>• relationships<br>2. NO tools are not validated<br>3. NO tools cover the mentoring process in its entirety

Professional Identity Formation

Two papers were drawn from current understanding of PIF. Like the other articles adopted in the assessment of mentoring, these reviews were papers published by the PMI team and were amongst the most up to date reviews on the subject.

Table B. Current reviews on PIF

Name	Publication	Lesson learnt
Sarraf-Yazdi et al (2021) (28)	A scoping review of professional identity formation in undergraduate medical education.	1. PIF is captured by changes in the RToP<br>2. PIF is and individualised process, person-specific, learning environment dependent and impacted the mentee's narratives, clinical competence and context and the<br>3. PIF evolves over time
Teo et al (2022) (29)	Assessing professional identity formation (PIF) amongst medical students in oncology and palliative medicine postings: a SEBA guided scoping review.	1. PIF remains poorly understood though the Krishna-Pisupati model does suggest that when the belief system is impacted by dyssynchrony, disharmony, resonance and or synchrony it results in changes to PIF<br>2. PIF changes depends on the community of practice and the socialisation process within it<br>3. PIF is also affected by the physicians' contextual considerations and narratives whilst their responses must be consistent with social expectations and practice<br>4. Future assessments must be<br>• Individualised<br>• Multi-sourced<br>• Longitudinal<br>• Use multiple forms of assessments<br>• Involve portfolios<br>• Overseen by the host organization

Stage 2c. Analysis

The lessons learnt from the study of these included reviews are summarised in Tables A and B.

Stage 2d. Combining

The key lessons learnt were combined.

Stage 2e. Supplementing the data

The findings were complemented by data revealing the efficacy of the RToP-based tool in

- Moral distress
  - Quek CWN, Ong RRS, Wong RSM, et al Systematic scoping review on moral distress among physicians BMJ Open 2022;12:e064029. doi: 10.1136/bmjopen-2022-064029
  - Ong, R.S.R., Wong, R.S.M., Chee, R.C.H. *et al.* A systematic scoping review moral distress amongst medical students. *BMC Med Educ* **22,** 466 (2022). https://doi.org/10.1186/s12909-022-03515-3

- Dignity
  - Quah ELY, Chua KZY, Lua JK, Wan DWJ, Chong CS, Lim YX, Krishna L. A Systematic Review of Stakeholder Perspectives of Dignity and Assisted Dying. J Pain Symptom Manage. 2022 Oct 13:S0885-3924(22)00924-1. doi: 10.1016/j.jpainsymman.2022.10.004. Epub ahead of print. PMID: 36244639.
  - Chua, K.Z.Y., Quah, E.L.Y., Lim, Y.X. *et al.* A systematic scoping review on patients' perceptions of dignity. *BMC Palliat Care* **21,** 118 (2022). https://doi.org/10.1186/s12904-022-01004-4

- Death and dying
  - Ho, C.Y., Lim, NA., Ong, Y.T. *et al.* The impact of death and dying on the personhood of senior nurses at the National Cancer Centre Singapore (NCCS): a qualitative study. *BMC Palliat Care* **21,** 83 (2022). https://doi.org/10.1186/s12904-022-00974-9
  - Chan, N.P.X., Chia, J.L., Ho, C.Y. *et al.* Extending the Ring Theory of Personhood to the Care of Dying Patients in Intensive Care Units. *Asian Bioethics Review* (2021). https://doi.org/10.1007/s41649-021-00192-0
  - Huang H, Toh RQE, Chiang CLL, Thenpandiyan AA, Vig PS, Lee RWL, Chiam M, Lee ASI, Baral VR, Krishna LKR. Title: Impact of Dying Neonates on Doctors' and Nurses' Personhood: A Systematic Scoping Review. J Pain Symptom Manage. 2021 Jul 13:S0885-3924(21)00425-5. doi: 10.1016/j.jpainsymman.2021.07.002. (in press)
  - Vig PS, Lim JY, Lee RW, Huang H, Tan XH, Lim WQ, Lim MB, Lee AS, Chiam M, Lim C, Baral VR. Parental bereavement–impact of death of neonates and children under 12 years on personhood of parents: a systematic scoping review. BMC palliative care. 2021 Dec;20(1):1-7.
  - Ho CY, Kow CS, Chia, JCH et al. The Impact of Death and Dying on the Personhood of Medical Students: A Systematic Scoping Review. *BMC Medical Education.* 2020; 20(516). Doi: 10.1186/s12909-020-02411-y
  - Kuek, J.T.Y., Ngiam, L.X.L., Kamal, N.H.A. *et al.* The impact of caring for dying patients in intensive care units on a physician's personhood: a systematic scoping review. *Philos Ethics Humanit Med* 15, 12 (2020). https://doi.org/10.1186/s13010-020-00096-1

- Changing concepts of self
  - Zhou X, Goh C, Chiam M, Krishna LKR. Painting and Poetry from a Bereaved Family and the Caring Physician *Journal of Pain and Symptom Management (2022) https://doi.org/10.1016/j.jpainsymman.2022.03.008*
  - Lim C, Zhou JXL, Woong NL et al. Addressing the needs of migrant workers in ICUs in Singapore. *Journal of Medical Education and Curricular Development*. 2020; 7:1-4. DOI: 10.1177/2382120520977190

Stage 3. Design of Tools – Semi-structured interviews and diaries design

The data from the SSRs in SEBA of CoPs and the socialisation were considered in tandem with Teo et al (29)'s recent review of PIF assessments. The conclusions drawn by the research team were reviewed by the expert team. This saw the design of semi-structured interview questionnaire and the mentoring diaries which serve to capture

- regnant social, cultural, political, institutional and personal considerations;
- social media, the hidden curriculum;
- practical factors and program, setting, speciality related issues and beliefs;
- codes of conduct, norms, and expectations;
- the team dynamics; the training approach;
- intra- and interprofessional practice
- the impact of the curriculum design (30, 31),
- the establishment of roles, responsibilities, and codes of conduct (31-33),
- the alignment of expectations (34, 35),
- participant selection (36),
- the employ of consistent teaching methods (32),
- role modelling and tutor selection (37),
- experiential learning (32),
- graded autonomy (38),
- allocated responsibility,
- assessment methods (31),
- institutional support,
- faculty development (39),
- guided reflections (40-42)
- remediation support (31, 43, 44)
- the privacy and support offered (31) by the learning environment (36, 45, 46).
- also characterise the socialization process (47, 48), the community of practice (49) and the Landscape of Communities (31), within the PMI which are posited by current theories to be key to PIF.

- features of resonance, synchrony, dyssynchrony, disharmony; and features of ‘sensitivity’, ‘events’, “willingness’, ‘judgement’ and ‘balance’.

Stage 4. Application of tools – Conducting semi-structured interviews and diaries

a. Semi-structured interviews

Eligible participants were peer-mentors in the PMI; specifically, peer-mentors who have completed mentoring programs as PMI mentees and who have been selected, trained and completed PMI mentoring programs as peer-mentors. Purposive sampling was conducted and email invitations containing a participant information sheet and consent form were sent out. The invitations stressed participant anonymity in the audio-recorded semi-structured interviews. The participants were also told of their right to withdraw from the study at any point and without prejudice. All participants provided written and verbal informed consent. After returning the signed consent forms, individual semi-structured interviews were arranged with each peer-mentor. These interviews took place in quiet offices to ensure privacy and facilitate in-depth exploration of personal beliefs, experiences, and practices. The semi-structured interviews were approximately 45min long and were conducted over the Zoom video conferencing platform. The semi-structured interviews were conducted between February and May 2021.

The two interviewers were briefed on the study aims and trained to conduct the interviews. As non-clinicians with no previous interactions nor dependent relationship with the participants, the trained interviewers sent out the email invites and arranged the meetings. This enhanced the participant anonymity from the research and expert teams. Audio recordings were transcribed verbatim using the NVivo 12 Software, anonymized and their integrity verified.

b. Mentoring diaries

Mentoring diaries were hosted on Google forms and were completed by all mentees and peer-mentors in the PMI on an ad-hoc basis between January to December 2021. The research team independently analyzed the interview transcripts using the SEBA methodology. The findings were compared with the findings of the semi-structured interviews to corroborate data.

c. Ethics approval

Ethics approval (CIRB Ref 2020/3056: Supporting Mentee Development: Milestones and Assessments in the Palliative Medicine Initiative) was obtained from the SingHealth Combined Institutional Review Board.

Stage 5. Split Approach

Concurrent use of Braun and Clarke (50)'s thematic analysis and Hsieh and Shannon (51)'s directed content analysis is consistent with the "traditional' SEBA methodology and serves to ensure a comprehensive, reproducible, transparent analysis (52-54). A grounded theory-based approach was not feasible, given the design and structure of the peer-mentoring program was heavily influenced by several theories on CoPs and PIF (1, 40, 55-59)

a. Thematic analysis

Using thematic analysis, two reviewers independently constructed 'codes' from the 'surface' meaning of the interview transcripts. These independent findings were discussed at online meetings to establish a common understanding of the 'codes'. Sandelowski and Barroso (3)'s approach to 'negotiated consensual validation' was used to achieve consensus on the initial codes and to establish a common coding framework and code book.

b. Directed content analysis.

Categories used directed content analysis were drawn from Cruess et al (55)'s "A schematic representation of the professional identity formation and socialization of medical students and residents: a guide for medical educators" and Ho (60)'s study entitled "The Impact of Death and Dying on the Personhood of Medical Students: A Systematic Scoping Review".

A common coding framework and code book for thematic analysis and another for content analysis were developed. Three reviewers independently coded and analysed the peer-mentor transcripts using thematic analysis and three reviewers independently analysed the data using content analysis. Subthemes and themes were developed upon collapsing the codes and larger inclusive concepts into even larger groups. Upon completion of the initial analysis, the research teams and expert team

discussed their findings to delineate specific characteristics of each theme carefully. Sandelowski and Barroso (3)'s approach to 'negotiated consensual validation' was used to achieve consensus on the final themes and subthemes from the first team and subcategories were agreed upon amongst the second team.

The process was repeated for the analysis of the mentoring diaries.

Stage 6. Jigsaw Perspective

The diary entries and data from the semi-structured interviews were considered separately. The similarities or areas of overlap between the categories and themes were then combined to create a bigger piece of the overlying data, with the combined themes and categories being referred to as themes/categories (52-54). To do this the Jigsaw Perspective employs Phases 4 to 6 from France, Uny (61)'s of Noblit and Hare (62)'s seven phases of meta-ethnography adaptation, to group the themes and the categories according to their focus. Similarities between the themes and categories allowed for the adoption of *reciprocal translation*, determining if the new data provided by the respective themes and categories was consistent with the subcategories and or subthemes within respective articles.

This process was repeated for the peer-mentor mentoring diaries.

The Jigsaw Perspective was then employed for a third time to combine the themes/categories drawn from analysis of the interview and diaries. This process of combining themes/categories from the semi-structured interviews and the mentoring diaries created domains.

Stage 7. Funneling Process

The themes/categories from the mentoring diaries and interviews were combined to create domains that frame the discussion (63).

References

1. Cruess RL, Cruess SR, Steinert Y. Medicine as a Community of Practice: Implications for Medical Education. Acad Med. 2018;93(2):185-91.
2. Deshpande A. The Use of Sociocultural Constructs in Cancer Screening Research Among African Americans. Cancer Control. 2009;16(3):10.
3. Sandelowski M, Barroso J. Handbook for synthesizing qualitative research. . New York: Springer Publishing Company; 2006.
4. Radha Krishna L, Renganathan Y, Tay K, Tan B, Chong J, Ching AH, et al. Educational roles as a continuum of mentoring's role in medicine – a systematic review and thematic analysis of educational studies from 2000 to 2018. BMC Med Educ 19, 439 (2019). Lalit Kumar Radha Krishna, Yaazhini Renganathan, Kuang Teck Tay, Benjamin Jia Xing Tan, Jia Yan Chong, Ann Hui Ching, Kishore Prakash, Nicholas Wei Sheng Quek, Rachel Huidi Peh, Annelissa Mien Chew Chin, David C. M. Taylor, Stephen Mason, Ravindran Kanesvaran & Ying Pin Toh.
5. Krishna L, Tay KT, Yap HW, Koh ZYK, Ng YX, Ong YT, et al. Combined novice, near-peer, e-mentoring palliative medicine program: A mixed method study in Singapore. Plos one. 2020;15(6):e0234322.
6. Krishna L, Toh YP, Mason S, Kanesvaran R. Mentoring stages: A study of undergraduate mentoring in palliative medicine in Singapore. PloS one. 2019;14(4):e0214643.
7. Krishna LKR, Tan LHE, Ong YT, Tay KT, Hee JM, Chiam M, et al. Enhancing Mentoring in Palliative Care: An Evidence Based Mentoring Framework. Journal of Medical Education and Curricular Development. 2020;7:2382120520957649.
8. Loo WTW, Ikbal MFBM, Wu JT, Wahab MT, Yeam CT, Ee HFM, et al. Towards a Practice Guided Evidence Based Theory of Mentoring in Palliative Care. J Palliat Care Med 2017;7(1).
9. Lee FQH, Chua WJ, Cheong CWS, Tay KT, Hian EKY, Chin AMC, et al. A Systematic Scoping Review of Ethical Issues in Mentoring in Surgery. Journal of Medical Education and Curricular Development. 2019;6:2382120519888915.
10. Cheong CWS, Chia EWY, Tay KT, Chua WJ, Lee FQH, Koh EYH, et al. A systematic scoping review of ethical issues in mentoring in internal medicine, family medicine and academic medicine. Advances in Health Sciences Education. 2019:1-25.
11. Tan B, Toh Y, Toh Y, Kanesvaran R, Krishna L. Extending mentoring in palliative medicine-systematic review on peer, near-peer and group mentoring in general medicine. Journal of Palliative Care & Medicine. 2017;7(06).
12. Hee JM, Yap HW, Ong ZX, Quek SQM, Toh YP, Mason S, et al. Understanding the Mentoring Environment Through Thematic Analysis of the Learning Environment in Medical Education: a Systematic Review. Journal of general internal medicine. 2019:1-10.
13. Tan YS, Teo SWA, Pei Y, Sng JH, Yap HW, Toh YP, et al. A framework for mentoring of medical students: thematic analysis of mentoring programmes between 2000 and 2015. Advances in Health Sciences Education. 2018;23(4):671-97.
14. Chong JY, Ching AH, Renganathan Y, Lim WQ, Toh YP, Mason S, et al. Enhancing mentoring experiences through e-mentoring: a systematic scoping review of e-mentoring programs between 2000 and 2017. Advances in Health Sciences Education. 2020;25(1):195-226.
15. Lim SYS, Koh EYH, Tan BJX, Toh YP, Mason S, Krishna LKR. Enhancing geriatric oncology training through a combination of novice mentoring and peer and near-peer mentoring: A thematic analysis ofmentoring in medicine between 2000 and 2017. J Geriatr Oncol. 2020;11(4):566-75.
16. Ng YX, Koh ZYK, Yap HW, Tay KT, Tan XH, Ong YT, et al. Assessing mentoring: A scoping review of mentoring assessment tools in internal medicine between 1990 and 2019. PLOS ONE. 2020;15(5):e0232511.
17. Chua WJ, Cheong CWS, Lee FQH, Koh EYH, Toh YP, Mason S, et al. Structuring Mentoring in Medicine and Surgery. A Systematic Scoping Review of Mentoring Programs Between 2000 and 2019. Journal of Continuing Education in the Health Professions. 2020;40(3):158-68.
18. Kow CS, Teo YH, Teo YN, Chua KZY, Quah ELY, Kamal N, et al. A systematic scoping review of ethical issues in mentoring in medical schools. BMC Med Educ. 2020;20(1):246.
19. Hee J, Toh YL, Yap HW, Toh YP, Kanesvaran R, Mason S, et al. The Development and Design of a Framework to Match Mentees and Mentors Through a Systematic Review andThematic Analysis of Mentoring Programs Between 2000 and 2015. Mentoring & Tutoring: Partnership in Learning. 2020;28(3):340-64.

20. Chia EWY, Tay KT, Xiao S, Teo YH, Ong YT, Chiam M, et al. The Pivotal Role of Host Organizations in Enhancing Mentoring in Internal Medicine: A Scoping Review. Journal of Medical Education
Curricular Development. 2020;7:2382120520956647.
21. Sng J, Pei Y, Toh Y, Peh T, Neo S, Krishna L. Mentoring relationships between senior physicians and junior doctors and/or medical students: A thematic review. Medical Teacher. 2017;39(866-875).
22. Krishna LKR, Tan LHE, Ong YT, Tay KT, Hee JM, Chiam M, et al. Enhancing Mentoring in Palliative Care: An Evidence Based Mentoring Framework. J Med Educ Curric Dev. 2020;7:2382120520957649.
23. Toh R, Koh K, Lua J, Wong R, Quah E, Panda A, et al. The role of mentoring, supervision, coaching, teaching and instruction on professional identity formation: a systematic scoping review. BMC Med Educ. 2022;22(531).
24. Chia EWY, Tay KT, Xiao S, Teo YH, Ong YT, Chiam M, et al. The Pivotal Role of Host Organizations in Enhancing Mentoring in Internal Medicine: A Scoping Review. J Med Educ Curric Dev. 2020;7:2382120520956647.
25. Goh S, Wong R, Quah E, Chua K, Lim W, Ng A, et al. Mentoring in palliative medicine in the time of covid-19: a systematic scoping review. BMC Medical Education. 2022;11(1):1-5.
26. Hee JM, Yap HW, Ong ZX, Quek SQM, Toh YP, Mason S, et al. Understanding the Mentoring Environment Through Thematic Analysis of the Learning Environment in Medical Education: a Systematic Review. J Gen Intern Med. 2019;34(10):2190-9.
27. Ng YX, Koh ZYK, Yap HW, Tay KT, Tan XH, Ong YT, et al. Assessing mentoring: A scoping review of mentoring assessment tools in internal medicine between 1990 and 2019. PloS one. 2020;15(5):e0232511-e.
28. Sarraf-Yazdi S, Teo Y, How A, Teo Y, Goh S, Kow C, et al. A scoping review of professional identity formation in undergraduate medical education. Journal of general internal medicine. JGIM. 2021;36(11):3511-21.
29. Teo K, Teo M, Pisupati A, Ong R, Goh C, Seah C, et al. Assessing professional identity formation (PIF) amongst medical students in Oncology and Palliative Medicine postings: a SEBA guided scoping review. BMC Palliat Care. 2022;21(200).
30. Jarvis-Selinger S. Residents' views of the role of classroom-based learning in graduate medical education through the lens of academic half days. Acad Med. 2015;90(4):532-8.
31. Cruess SR, Cruess RL, Steinert Y. Supporting the development of a professional identity: General principles. Med Teach. 2019;41(6):641-9.
32. Cruess S, Cruess R. Teaching professionalism - Why, What and How. Facts Views Vis Obgyn. 2012;4(4):259-65.
33. Cruess S, Johnston S, Cruess R. Professionalism for medicine: opportunities and obligations. Med J Aust. 2002;177(4):208-11.
34. Steinauer J, O'Sullivan P, Preskill F, Ten Cate O, Teherani A. What Makes "Difficult Patients" Difficult for Medical Students? Acad Med. 2018;93(9):1359-66.
35. Stubbing E, Helmich E, Cleland J. Medical student views of and responses to expectations of professionalism. Med Educ. 2019;53(10):1025-36.
36. Boudreau J, Cruess S, Cruess R. Physicianship: Educating for professionalism in the post-Flexnarian era. Perspect Biol Med. 2011;54(1):89-105.
37. Passi V, Johnson S, Peile E, Wright S, F H, Johnson N. Doctor role modelling in medical education: BEME Guide No. 27. Med Teach. 2013;35(9):e1422-e36.
38. Sternszus R, Boudreau J, Cruess R, Cruess S, Macdonald M, Steinert Y. Clinical Teachers' Perceptions of Their Role in Professional Identity Formation. Acad Med. 2020;95(10):1594-9.
39. Mak-van der Vossen M, Peerdeman S, van Mook W, Croiset G, Kusurkar R. Assessing professional behaviour: Overcoming teachers' reluctance to fail students. BMC Res Notes. 2014;7(368).
40. Jarvis-Selinger S, MacNeil KA, Costello GR, Lee K, Holmes CLJAM. Understanding professional identity formation in early clerkship: a novel framework. 2019;94(10):1574-80.
41. Steinauer J, Teherani A, Mangini R, Chien J, ten Cate O, O'Sullivan P. Characterizations of motivation and identity in medical students' reflections about challenging patient interactions. Med Teach. 2019;41(10):1178-83.
42. Jarvis-Selinger S, Pratt DD, Regehr G. Competency Is Not Enough: Integrating Identity Formation Into the Medical Education Discourse. Academic Medicine. 2012;87(9):1185-90.
43. Cruess RL, Cruess SR, Boudreau JD, Snell L, Steinert Y. Reframing medical education to support professional identity formation. Acad Med. 2014;89(11):1446-51.

44. Steinert Y, Cruess R, Cruess S, Boudreau J, Fuks A. Faculty development as an instrument of change: a case study on teaching professionalism. Acad Med. 2007;82(11):1057-64.
45. Berkhout J, Helmich E, Teunissen P, van der Vleuten C, Jaarsma A. Context matters when striving to promote active and lifelong learning in medical education. Med Educ. 2018;52(1):34-44.
46. Monrouxe L, Chandratilake M, Gosselin K, Rees C, Ho M. Taiwanese and Sri Lankan students' dimensions and discourses of professionalism. Med Educ. 2017;51(7):718-31.
47. Cruess R, Cruess S, Boudreau J, Snell L, Steinert Y. A schematic representation of the professional identity formation and socialization of medical students and residents: a guide for medical educators. Acad Med. 2015;90(6):718-25.
48. Papadakis M, Paauw D, Hafferty F, Shapiro J, Byyny R. Alpha Omega Alpha Honor Medical Society Think Tank. Perspective: the education community must develop best practices informed by evidence-based research to remediate lapses of professionalism. Acad Med. 2012;87(12):1694-8.
49. Cruess R, Cruess S, Steinert Y. Medicine as a Community of Practice: Implications for Medical Education. Acad Med. 2018;93(2):185-91.
50. Braun V, Clarke V. Using thematic analysis in psychology. . Qualitative Research in Psychology 2006;3(2):77-101.
51. Hsieh H-F, Shannon SE. Three Approaches to Qualitative Content Analysis. Qualitative Health Research. 2005;15(9):1277-88.
52. Kamal NHA, Tan LHE, Wong RSM, Ong RRS, Seow REW, Loh EKY, et al. Enhancing education in Palliative Medicine: the role of Systematic Scoping Reviews. Palliative Medicine & Care: Open Access. 2020;7(1):1-11.
53. Ong RRS, Seow REW, Wong RSM. A Systematic Scoping Review of Narrative Reviews in Palliative Medicine Education. Palliative Medicine & Care: Open Access. 2020;7(1):1-22.
54. Mah ZH, Wong RSM, Seow REW, Loh EKY, Kamal NHA, Ong RRS, et al. A Systematic Scoping Review of Systematic Reviews in Palliative Medicine Education. Palliative Medicine & Care: Open Access. 2020;7(1):1-12.
55. Cruess RL, Cruess SR, Boudreau JD, Snell L, Steinert Y. A schematic representation of the professional identity formation and socialization of medical students and residents: a guide for medical educators. Acad Med. 2015;90(6):718-25.
56. Sawatsky AP, Santivasi WL, Nordhues HC, Vaa BE, Ratelle JT, Beckman TJ, et al. Autonomy and professional identity formation in residency training: a qualitative study. Medical education. 2020;54(7):616-27.
57. Barnhoorn PC, Houtlosser M, Ottenhoff-de Jonge MW, Essers G, Numans ME, Kramer AWM. A practical framework for remediating unprofessional behavior and for developing professionalism competencies and a professional identity. Med Teach. 2019;41(3):303-8.
58. Mak-van der Vossen M, Teherani A, van Mook W, Croiset G, Kusurkar RA. How to identify, address and report students' unprofessional behaviour in medical school. Medical Teacher. 2020;42(4):372-9.
59. Wald HS, Anthony D, Hutchinson TA, Liben S, Smilovitch M, Donato AA. Professional identity formation in medical education for humanistic, resilient physicians: pedagogic strategies for bridging theory to practice. Academic Medicine. 2015;90(6):753-60.
60. Ho CY, Kow CS, Chia CHJ, Low JY, Lai YHM, Lauw S-K, et al. The impact of death and dying on the personhood of medical students: a systematic scoping review. BMC Medical Education. 2020;20(1):516.
61. France EF, Uny I, Ring N, Turley RL, Maxwell M, Duncan EAS, et al. A methodological systematic review of meta-ethnography conduct to articulate the complex analytical phases. BMC Med Res Methodol. 2019;19(1):35.
62. Noblit GW, Hare RD. Meta-ethnography: synthesizing qualitative studies. California: Sage Publications; 1988.
63. Lingard L, Watling C. It's a Story, Not a Study: Writing an Effective Research Paper. Academic Medicine. 2016;91(12):e12
